# Complete Genome Sequence of *Bradyrhizobium diazoefficiens* USDA 122, a Nitrogen-Fixing Soybean Symbiont

**DOI:** 10.1128/genomeA.01743-16

**Published:** 2017-03-02

**Authors:** Masayuki Sugawara, Takahiro Tsukui, Takakazu Kaneko, Yoshiyuki Ohtsubo, Shusei Sato, Yuji Nagata, Masataka Tsuda, Hisayuki Mitsui, Kiwamu Minamisawa

**Affiliations:** a Graduate School of Life Sciences, Tohoku University, Sendai, Japan; b Faculty of Engineering Life Sciences, Kyoto Sangyo University, Kyoto, Japan; c Kazusa DNA Institute, Kisarazu, Chiba, Japan

## Abstract

We report the complete genome sequence of *Bradyrhizobium diazoefficiens* USDA 122, a nitrogen-fixing soybean symbiont. The genome consists of a 9.1 Mb circular chromosome, and 8,551 coding sequences (CDSs) were predicted on the genome. The sequence will provide insight into the evolution of rhizobial genome, and the symbiotic compatibility with host plants.

## GENOME ANNOUNCEMENT

*Bradyrhizobium diazoefficiens* (reclassified from *B. japonicum*) is a nitrogen-fixing symbiont for several legumes. The complete genome of USDA 110^T^ was reported in 2002 (1). Comparative genomics indicated the presence of genomic variations between USDA 122 and USDA 110^T^, even though their 16S rRNA gene sequences are identical (2). Furthermore, symbiotic compatibility with *Rj2*-soybean plants differs between the two strains (3, 4). Thus, we determined whole-genome sequence of USDA 122.

The genome of USDA 122 was sequenced using 454 GS-FLX Titanium (Roche, Basel, Switzerland) and Illumina MiSeq (Illumina, San Diego, CA, USA), in combination with BAC and cosmid end sequencing. A fragmented genome library was constructed by the Covaris S2-A system (Covaris, Woburn, MA, USA) for 454 GS-FLX Titanium. As for MiSeq paired-end reads, the published data (DDBJ Sequence Read Archive: DRX022752) was used for this study (5). The paired-end reads were trimmed by ShortReadManager (6), in which 21-mers occurring more than two times were regarded as valid. The BAC and cosmid libraries were constructed as previously reported (7). The BAC library (Bj122b; 2,688 clones) and the cosmid library (Bj122c; 2,304 clones) contained inserts of 64.9 kb and 26.6 kb in average length, respectively. The sequences at both ends of the clones from the Bj122b and Bj122c were analyzed with the Sanger method using a BigDye-terminator cycle sequencing kit and a 3730xl Sequencer (Life Technologies, Foster City, CA, USA). Those reads [1,691,796 reads from 454 GS-FLX (738 Mb), 2,075,228 reads from MiSeq (410 Mb), and end-sequences of Bj122c (1,979 pairs) and Bj122b (1,661pairs)] were assembled by using Newbler version 2.8 (Roche), and resulted in the generation of 275 contigs and 31 scaffolds. The finishing was facilitated by GenoFinisher and AceFileViewer (6), in which predicted contig adjacencies were confirmed by PCR or determined by combinatorial PCR, and gap sequences were determined by *in silico* analysis or, if necessary, sequencing of the PCR products. The finished sequence was validated by FinishChecker (6). The genome sequence was annotated using the NCBI Prokaryotic Genome Annotation Pipeline (8), and the result was manually inspected with respect to positions of start codons for predicted open reading frames (ORFs) using the Microbial Genome Annotation Pipeline (MiGAP; http://www.migap.org/) and GenomeMatcher (9).

The genome of USDA 122 consists of a single chromosome (9,136,536 bp, 64.0% G+C). A total of 8,551 protein-coding genes, 51 tRNAs, and three rRNAs were predicted on the genome. The average nucleotide identity (10) and the DNA-DNA hybridization (11) values between the genomes of USDA 122 and 110^T^ were 98.5% and 89.4%, respectively. A dot plot analysis revealed that USDA 122 genome possesses a strong collinearity with USDA 110^T^ genome with a large inversion. The results of pan-genome analysis based on protein clustering revealed that the predicted 1,069 gene products (12.5%) did not show high similarity (<70% amino acid sequence identity) to any genes of USDA 110^T^.

### Accession number(s).

The genome sequence of USDA 122 and the end sequences of BAC and cosmid libraries have been deposited at DDBJ/EMBL/GenBank under the accession numbers CP013127, FT932078 to FT936035, and FT936036 to FT939357, respectively.
